# Application of Pulsed Electric Fields to Enhance the Digestive Bioaccessibility of *Chlorella vulgaris* Protein Fractions

**DOI:** 10.3390/foods15213882

**Published:** 2026-10-29

**Authors:** Luís Machado, Serena Carpentieri, Raquel F. S. Gonçalves, Joana T. Martins, Maria Silva, João Martinho, José A. Teixeira, Giovanna Ferrari, Ricardo N. Pereira, Gianpiero Pataro

**Affiliations:** 1 CEB—Centre of Biological Engineering, University of Minho, 4710-057 Braga, Portugalraquel.goncalves@ceb.uminho.pt (R.F.S.G.); joanamartins@ceb.uminho.pt (J.T.M.); jateixeira@deqb.uminho.pt (J.A.T.); 2 Department of Industrial Engineering, University of Salerno, Via Giovanni Paolo II, 132, 84084 Fisciano, SA, Italy; scarpentieri@unisa.it (S.C.); gferrari@unisa.it (G.F.); 3 LABBELS—Associate Laboratory, Braga, Guimarães, Portugal; 4 ProdAl Scarl, University of Salerno, Via Giovanni Paolo II, 132, 84084 Fisciano, SA, Italy

**Keywords:** Pulsed Electric Fields, *Chlorella vulgaris*, gastrointestinal digestion, amino acids profile, protein bioaccessibility

## Abstract

Microalgae are widely used as dietary supplements. Despite their recognized nutritional value, it remains unclear whether consumption of microalgal biomass provides sufficient nutrient bioaccessibility to meet human dietary needs. In this work, the microalga *Chlorella vulgaris* CCAP 211/11B was cultivated and subjected to Pulsed Electric Fields (PEF) pre-treatment (20 kV·cm^−1^, 100 kJ·kg^−1^). The effects of PEF pretreatment were evaluated by assessing protein bioaccessibility throughout the different gastrointestinal (GI) digestion phases, as well as the amino acid profile and cytotoxicity in Caco-2 cells. During the oral and gastric phases, no significant differences (*p* > 0.05) in protein release were observed between PEF-treated and untreated biomass. At the final intestinal stage, however, protein release was 21.7% in PEF-treated biomass compared with 14.7% in untreated samples, corresponding to a 47% increase in protein bioaccessibility due to PEF pretreatment. Intrinsic fluorescence spectroscopy did not clearly discriminate treatments due to the dominant signal from digestive enzymes. Beyond improving protein availability, PEF-treated *C. vulgaris* exhibited no cytotoxic effects, confirming the safety of this processing approach for food applications aimed at enhancing protein uptake. Simulated GI digestion of both untreated and PEF-treated samples resulted in comparable amino acid profiles, with tyrosine, methionine, leucine, and lysine detected at the highest relative levels. Overall, these findings demonstrate that PEF treatment represents a promising strategy to enhance protein bioaccessibility in *Chlorella vulgaris*, thereby strengthening its potential as a functional and sustainable food supplement.

## 1. Introduction

Microalgae are photosynthetic microorganisms capable of growing in a wide range of environments. They are easy to cultivate, require less land than conventional food crops [1], and exhibit exceptionally high photosynthetic efficiency [2]. This remarkable efficiency has driven growing interest in microalgae as a sustainable and versatile source of high-value biomolecules, including proteins, lipids, carbohydrates, carotenoids, pigments, and vitamins. These compounds show significant potential for applications across various sectors, including food, feed, pharmaceuticals, cosmetics, and renewable energy [3,4,5].

In the European Union, the regulatory status of *Chlorella* spp. has also been established through the consultation process under Article 4 of Regulation (EU) 2015/2283. In a consultation concluded in 2022, *Chlorella* spp., including *C. vulgaris*, were considered not to fall under the scope of the Novel Food Regulation based on evidence of a history of consumption as food or food supplements in the EU before 15 May 1997. The consultation also indicates that dried *Chlorella* biomass can be used as a food material, including as a component of products such as bakery products, pasta, and beverages. This established food-use status further supports the relevance of *C. vulgaris* as a candidate for food applications and for the development of processing strategies aimed at improving the bioaccessibility of its nutrients.

Despite claimed advantages, the valuable intracellular compounds are confined within the cytoplasm and organelles and are protected by a complex cell envelope composed of membranes and a rigid cell wall. This structural barrier represents a major challenge for efficient mass transfer and extraction of intracellular components [6]. In green algae (*Chlorophyta*), such as *Chlorella vulgaris*, the cell wall, located outside the semipermeable cytoplasmic membrane, is primarily composed of polysaccharides, particularly cellulose and hemicelluloses, which may constitute up to 10% of the dry biomass [7]. Since the human gastrointestinal (GI) tract lacks the enzymes required to degrade these polysaccharides, the direct consumption of intact microalgae limits the bioavailability of their nutrients [8,9,10]. Nonetheless, multiple studies have demonstrated that applying mild cell disruption pretreatments to microalgae with complex cell wall structures significantly enhances the bioaccessibility of intracellular nutrients, without adversely affecting their nutritional quality or functional properties [11]. In this context, PEF technology has emerged as an efficient and scalable cell disintegration method capable of gently permeabilizing the envelope of different microalgae, including *C. vulgaris* [6,12,13].

During PEF treatment, the algae suspension is positioned between two metal electrodes of a treatment chamber and exposed to intense electric fields (10–40 kV/cm). These fields are delivered as repetitive short pulses, on the order of microseconds, which induce membrane electroporation, thereby enhancing cell permeability [13,14]. This mild approach facilitates the release of intracellular compounds, while preserving the integrity of sensitive biocompounds [12,13,15].

Although PEF is highly effective in permeabilizing the cytoplasmic membrane, it often leaves the rigid outer cell wall largely intact, particularly in species with “hard-structured” walls, thereby necessitating complementary strategies to enhance compound recovery [13]. One promising approach involves the sequential application of PEF and enzymatic hydrolysis [6]. Several studies have shown that using PEF as a pretreatment prior to enzymatic hydrolysis can significantly enhance protein release from various microalgae, including *Chlorella vulgaris*, *Palmaria palmata*, and *Scenedesmus almeriensis*, thereby improving the overall efficiency of the hydrolysis process [16,17,18]. This enhancement is thought to arise because electroporation induced by PEF not only facilitates the release of intracellular compounds, including low-molecular-weight proteins, but also increases enzyme accessibility to the intracellular space, enabling high-molecular-weight proteins to be cleaved into smaller peptides that can diffuse more readily [6]. However, research on the integration of PEF and enzymatic hydrolysis remains limited, and, to date, no studies have specifically investigated the combination of PEF pretreatment of microalgal cells with the enzymatic activities involved in GI digestion. In addition, the potential cytotoxic effects arising from the direct contact between metal electrodes and microalgal biomass remain largely undisclosed.

Nevertheless, recent advances in food biotechnology have made it possible to accurately simulate human GI digestion in vitro [19]. This approach replicates the key physiological conditions of the digestion process, from the oral phase through the gastric and intestinal phases, thereby enabling the creation of a comprehensive digestibility profile for microalgae. By the combination of PEF pretreatment of microalgal biomass with in vitro GI digestion, it becomes possible to evaluate how PEF technology influences digestibility of microalgae cells and the bioaccessibility of its proteins fraction.

Therefore, this study aims to investigate the impact of PEF treatment on the digestibility of *C. vulgaris* during simulated in vitro GI digestion, as well as on the bioaccessibility of its protein fractions following digestion. The extent and quality of protein release throughout the different GI digestion phases were evaluated by quantifying the total protein content, supported by fluorescence analysis and electrophoretic profiling of protein fractions using SDS-PAGE. Finally, the effect of the digested microalgal biomass on amino acid composition and viability of Caco-2 human intestinal cells was assessed.

## 2. Materials and Methods

### 2.1. Microalgae Cultivation and Harvesting

The microalgae *C. vulgaris* CCAP 211/11B was purchased from the Culture Collection of Algae & Protozoa (CCAP, Oban, UK). Cultivation was performed at the Centre of Biological Engineering, University of Minho (Braga, Portugal), in a 16 L flat panel photobioreactor (73 cm height × 5 cm width) with a working volume of 10 L. The aeration stream was enriched with 1% (v·v^−1^) CO_2_ and filtered through a 0.2 μm PTFE membrane filter (Midisart 2000, Sartorius, Göttingen, Germany) prior to entering the reactor. The culture was maintained under continuous illumination at an intensity of 175 μmol photons m^−2^·s^−1^ for 12 days. A general-purpose growth medium containing all essential macro- and micronutrients required for optimal microalgae growth was employed, as described by Machado et al. [20].

After cultivation, the *C. vulgaris* biomass was harvested by centrifugation at 1489× *g* for 10 min using a refrigerated centrifuge (Multifuge X3R, Thermo Scientific, Waltham, MA, USA). The resulting pellet was subsequently freeze-dried to remove residual moisture. The lyophilized biomass was then sent to the laboratory of ProdAl Scarl, (University of Salerno, Italy), for further processing using PEF equipment.

### 2.2. Preparation of C. vulgaris Suspension

A *C. vulgaris* suspension was prepared at a concentration of 1% (w·v^−1^) in a citric acid–Na_2_HPO_4_ buffer. This concentration was selected to facilitate adjustment and control of the electrical conductivity during PEF treatment while providing a suitable biomass concentration for the subsequent characterization assays performed in this study. The buffer solution was obtained by mixing 0.1 M citric acid and 0.2 M Na_2_HPO_4_ to achieve a final pH of 7.0. The electrical conductivity of the microalgal suspension was then adjusted by adding distilled water up to 2 mS·cm^−1^ at 25 °C using a conductivity meter (HI 9033, Hanna Instruments, Milan, Italy).

### 2.3. PEF Pre-Treatment

PEF pre-treatments of *C. vulgaris* suspension were performed using a bench-scale continuous-flow PEF unit, as detailed in a previous study [12]. The suspensions were circulated through the PEF unit at a controlled flow rate of 2 L·h^−1^ using a peristaltic pump (PU-2080, Jasco Europe, Cremella, Italy). The PEF treatment zone consisted of four co-field treatment chambers, each with an inner radius of 1.5 mm and a 4 mm electrode gap. These chambers were hydraulically connected in series and arranged into two modules (two chambers per module). The inlet temperature of samples to each module was controlled using a stainless-steel coiled tube submerged in a water heating bath (Thermo Haake DC 10, Henco Srl, Venice, Italy) set at 25 °C. The system was powered by a high-voltage pulsed power generator (20 kV–100 A; Diversified Technology Inc., Bedford, WA, USA), capable of delivering monopolar square pulses. The equipment allows independent adjustment of the applied voltage (0–20 kV), pulse width (1–10 μs), and pulse repetition rate (1–1000 Hz), with a maximum average power of 25 kW. The maximum electric field strength (E, in kV·cm^−1^) and total specific energy input (W_T_, in kJ·kg^−1^ suspension) were determined following the method described by Postma et al. [21]. Product temperatures at the inlet and outlet of each PEF module were continuously monitored using T-type thermocouples.

In this study, all experiments were conducted under fixed operating conditions: an electric field strength of 20 kV·cm^−1^, a total specific energy input of 100 kJ·kg^−1^ of suspension (equivalent to approximately 0.028 kWh·kg^−1^ of treated suspension), and a pulse width of 5 μs.

These parameters were previously identified as optimal for maximizing the release of carbohydrates and water-soluble proteins from *C. vulgaris* cells while minimizing treatment severity [12]. Under these conditions, the temperature increase at the exit of each module due to the Joule effect never exceeded 10 °C.

Both untreated and treated microalgal suspensions were collected in a 500 mL flask at the PEF outlet and immediately cooled in an ice-water bath to room temperature prior to in vitro GI digestion. All treatments were performed in triplicate.

### 2.4. In Vitro GI Digestion

The in vitro GI digestion procedure was adapted from the standardized COST INFOGEST consensus method [19], with minor modifications. Briefly, 20 mL of *C. vulgaris* paste (PEF-treated or untreated) was mixed with 20 mL of simulated salivary fluid (SSF), consisting of 5 g·L^−1^ of α-amylase, 0.117 g·L^−1^ of NaCl, 0.149 g·L^−1^ of KCl, 2.1 g·L^−1^ of Na_2_CO_3_, and 0.074 g·L^−1^ of CaCO_3_. The oral phase was conducted for 30 s under continuous agitation at 37 °C.

Following the oral phase, the entire mixture was combined at a 1:1 (v:v) ratio with simulated gastric fluid (SGF), composed of 3.2 g·L^−1^ pepsin and 2 g·L^−1^ NaCl. The pH was adjusted to 3 using 1 mol·L^−1^ HCl, and the gastric digestion proceeded for 2 h at 37 °C with constant agitation. Subsequently, the intestinal phase was initiated by mixing the gastric digest 1:1 (v:v) with simulated intestinal fluid (SIF), containing 5 g·L^−1^ pancreatin, 0.2 g·L^−1^ CaCO_3_, 0.2 g·L^−1^ CaCl_2_, 1.75 g·L^−1^ NaCl, and 25 g·L^−1^ bile salts. The pH was adjusted to 7 using 1 mol·L^−1^ NaOH, and the mixture was incubated for 2 h at 37 °C, followed by an additional 30 min to ensure complete digestion. Alongside the PEF-treated and untreated *C. vulgaris* samples, a blank digestion (using water instead of microalgae paste) was performed to account for background enzyme activity.

Throughout the digestion process, aliquots were collected to assess the release of proteins from *C. vulgaris* cells. In total, 6 samples were obtained: one after the oral phase; two during the gastric phase, and three during the intestinal phase. All samples were centrifuged at 18,440× *g* for 5 min using a benchtop centrifuge (5417R Centrifuge, Eppendorf, Hamburg, Germany), and the resulting supernatants were analyzed following digestion.

### 2.5. Protein Release and Detection After In Vitro GI Digestion

The samples collected after each stage of the in vitro simulated GI digestion, namely the oral, gastric, and intestinal phases, were centrifuged at 18,440× *g* for 15 min at room temperature (23 °C) to remove cell debris. The resulting supernatants were then analyzed using the Lowry protein quantification method, fluorescence spectroscopy, and SDS-PAGE to evaluate the total protein release from *C. vulgaris* cells.

The performance of these analytical techniques was compared in terms of detection efficiency, sensitivity to detect protein availability, and overall suitability for assessing microalgal proteins after PEF processing.

#### 2.5.1. Protein Quantification by Lowry Method

The total protein content released during each digestion phase was determined using the Lowry method, as previously described by Machado et al. [20]. Briefly, 500 μL of the digested sample, previously diluted tenfold, was mixed with 1.25 mL of Lowry reagent and incubated in the dark at room temperature for 10 min. Subsequently, 250 μL of Folin reagent was added, and the mixture was incubated for an additional 30 min under the same conditions. Absorbance was then measured at 750 nm using a UV/Vis spectrophotometer (V-650, Jasco Inc., Easton, MD, USA).

Serial dilutions of a Bovine Serum Albumin (BSA) solution (500 mg·L^−1^) were prepared to generate a standard curve for protein concentration quantification (Equation (1)):

*Abs*_750nm_ = 0.0016 × *BSA* (mg·L^−1^) + 0.0511; *R*^2^ = 0.9922. (1)

The results were expressed as protein yield (Yp), calculated by normalizing the amount of released protein to the total protein content of *C. vulgaris* cells.

#### 2.5.2. Fluorescence Analysis

Intrinsic fluorescence of the digested samples was recorded using a spectrofluorometer (Aqualog, Horiba Jobin Yvon, Longjumeau, France) equipped with a quartz cuvette (High Precision Cell, 10 × 2 mm optical path; Hellma Analytics, Baden-Württemberg, Germany). The excitation wavelength was set to 280 nm to detect protein-specific fluorescence arising from tryptophan (Trp) and tyrosine (Tyr). At this wavelength, the aromatic amino acids within the protein structure absorb the light and subsequently emit fluorescence at longer wavelengths, typically around 330 nm [22,23].

The fluorescence spectra were further processed by calculating the integrated peak area of each spectrum to facilitate data analysis. The reported intensity values were scaled down by a factor of 10^4^ relative to the measured data.

#### 2.5.3. Electrophoretic Profile of Protein Fractions (SDS-PAGE)

For the SDS-PAGE analysis, the protocol for gel preparation and data interpretation followed the methodology previously described by Pereira et al. [24]. Both untreated (control) and PEF-treated samples were analyzed and directly compared. Additionally, a separate gel containing only the GI enzymes was run as a blank to distinguish the protein bands originating from the digestive enzymes and from those released by the microalgae.

### 2.6. Caco-2 Cell Culture and Viability Assay

The human colon carcinoma Caco-2 cell line (American Type Culture Collection, ATCC) was cultured in Dulbecco’s modified Eagle’s medium (Lonza, Basel, Switzerland) supplemented with 10% (v·v^−1^) fetal bovine serum (Merck, Darmstadt, Germany), 1% (v·v^−1^) penicillin/streptomycin (Sigma-Aldrich, St. Louis, MO, USA) and 1% (v·v^−1^) non-essential amino acids (Lonza, Basel, Switzerland). Cells were maintained at 37 °C in a humidified atmosphere containing 5% CO_2_. Cell viability was assessed using the 3-(4,5-dimethylthiazol-2-yl)-2,5-diphenyltetrazolium bromide (MTT) assay, following the methodologies described by Gonçalves et al. and DeLoid et al. [25,26]. Briefly, cells were seeded in a 96-well plate at a density of 2 × 10^5^ cells/well and incubated for 24 h at 37 °C in 5% CO_2_. The culture medium was then replaced with 200 μL of fresh medium containing either untreated or PEF-treated digested *C. vulgaris* (micellar phase) at various dilution factors (i.e., 1:10, 1:15 and 1:20, v·v^−1^), followed by a 4 h incubation under the same conditions. After incubation, the medium was removed, and cells were gently washed with 200 μL of phosphate-buffered saline (PBS; Lonza, Basel, Switzerland). Subsequently, 100 μL of MTT solution (0.5 mg·mL^−1^ in PBS; Sigma-Aldrich, St. Louis, MO, USA) was added to each well and incubated for 3 h at 37 °C in a 5% CO_2_ humidified atmosphere.

The MTT solution was then removed, and 200 μL of dimethyl sulfoxide (Fisher Scientific, Fair Lawn, NJ, USA) was added to dissolve the formazan crystals formed by metabolically active cells. Cell viability was assessed spectrophotometrically by measuring absorbance at 570 nm with a reference wavelength of 630 nm and was calculated using Equation (2).

(2) Cell viability%=AbssampleAbscontrol×100

where Abs_sample_ and Abs_control_ correspond to the absorbance of wells containing Caco-2 cells with digested microalgal medium and Caco-2 cells with standard culture medium, respectively.

### 2.7. Chlorella Vulgaris Amino Acids Profile

The amino acid profile of the extracts obtained from both untreated and PEF-treated samples was analyzed using Ultra High-Performance Liquid Chromatography (UHPLC).

#### 2.7.1. Preparation of Derivatization Reagents and Eluents

A Shimadzu Nexera XR/RF-20AXS system (Shimadzu Corporation, Kyoto, Japan) equipped for pre-column automatic derivatization was used. In this system, the sample and derivatization reagents are mixed directly within the autosampler needle. All reagents were protected from light using aluminium foil and stored at 4 °C. The preparation details for all solutions, reagents, and mobile phases are provided in the Supplementary Materials (Table S1).

#### 2.7.2. Preparation of the Samples

A calibration curve was first prepared using standard solutions containing all amino acids. Subsequently, both the standards and the extracts obtained from non-treated and PEF-treated samples were filtered through a 0.22 μm filter. Then, 40 μL of each sample was transferred into a vial insert, followed by the addition of 40 μL of the internal standard (5 mg·L^−1^).

#### 2.7.3. Analytical Conditions

Amino acid quantification was performed using UHPLC on a Shimadzu Nexera XR/RF-20AXS system equipped with a Shim-pack^TM^ XR-ODSII column (2.2 μm, 100 mm × 3.0 mm), operated under a low-pressure gradient. The flow rate was set to 0.8 mL·min^−1^ with an injection volume of 1 μL. The column temperature was maintained at 40 °C, and the autosampler cooler was kept at 4 °C. Fluorescence detection was performed at λ_Ex_ = 350 nm and λ_Em_ = 450 nm, with the detector cell maintained at 25 °C.

### 2.8. Statistical Analysis

Results are presented as the mean ± standard deviation (SD) of three to four independent biological replicates (*n* = 3–4). Statistical analyses were performed using GraphPad Prism 9 software (version 9.3.1). A two-way analysis of variance (ANOVA) was conducted with a 95% confidence level, with a Tukey post hoc test. *t*-tests were also conducted when needed.

## 3. Results and Discussion

To evaluate protein bioaccessibility during GI digestion, intrinsic fluorescence spectroscopy and SDS-PAGE were employed to investigate structural modifications and changes in the protein profile, respectively. Protein concentration was subsequently determined using the Lowry assay. All analyses were performed on samples collected at different stages of the GI digestion process.

### 3.1. Protein Release During GI Digestion

**Figure 1:**
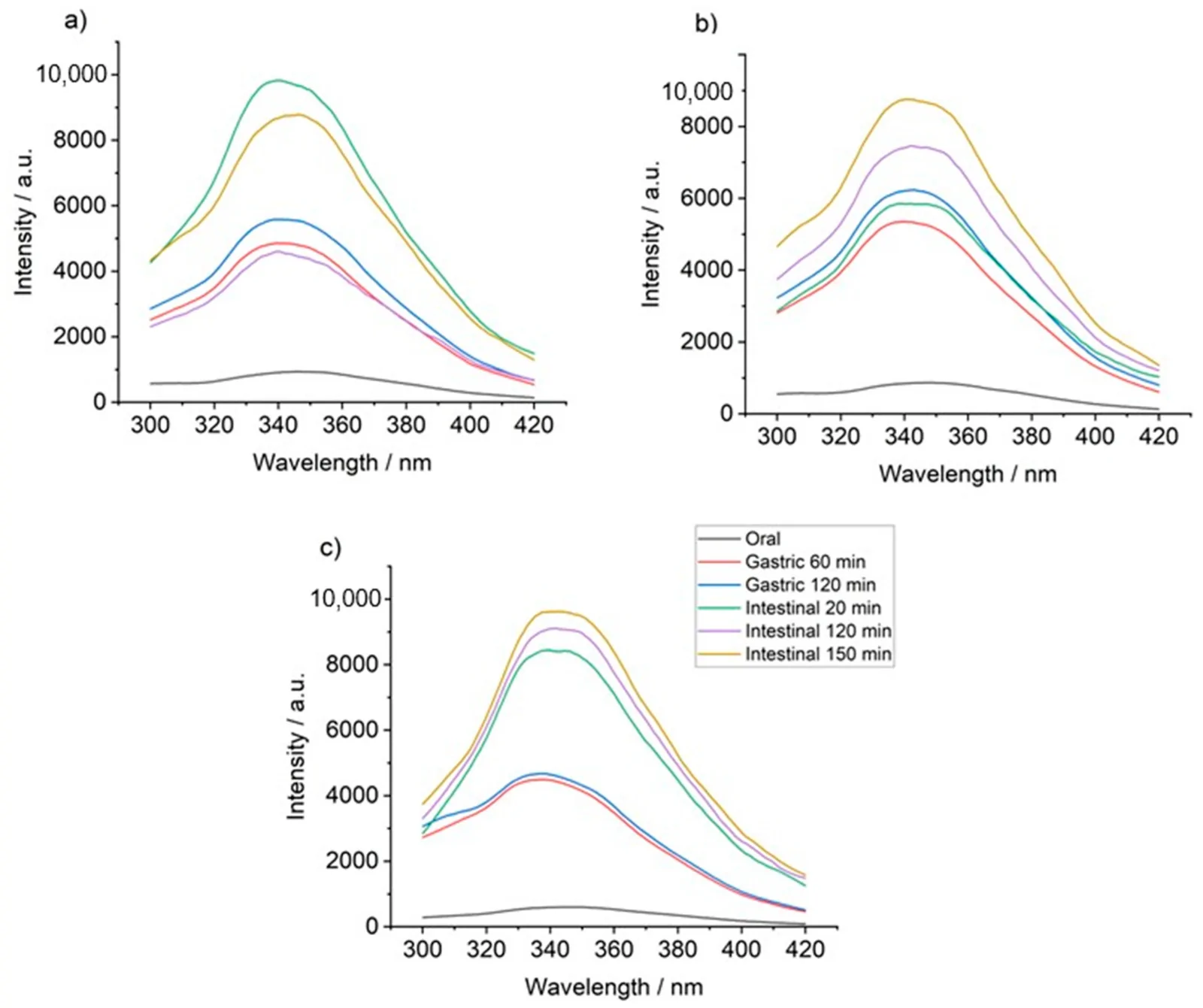
Intrinsic fluorescence spectra obtained at the different stages of GI digestion: (**a**) Control (without PEF pretreatment); (**b**) PEF-pretreated; and (**c**) GI enzymes.

The results of the intrinsic fluorescence analysis of the digested samples are presented in Figure 1. The spectra of the GI enzymes (Figure 1c) were used for baseline correction to eliminate spectral interferences arising from the enzymes added during microalgae digestion. It can be noted that the intensity of GI enzyme spectra increased with the number of enzymes sequentially added during the digestion process. For example, lower fluorescence was observed during the oral phase (α-amylase only) compared to the gastric (pepsin) or intestinal (pancreatin) phases.

During the oral phase, the lowest fluorescence intensity was recorded under all conditions. This may be associated with three factors: (i) the limited amount of GI enzymes present at the initial stage of digestion; (ii) the low content of aromatic amino acid residues in α-amylase, which contains only two tryptophan (Trp) residues—primarily responsible for protein intrinsic fluorescence [23]; and/or (iii) the limited effect of oral enzymes on *C. vulgaris*. As digestion progressed, fluorescence intensity increased. It is important to note that most of the detected fluorescence signals likely originated from the GI enzymes. In fact, the fluorescence spectra of the enzyme controls exhibited patterns similar to those observed for both control and PEF-pretreated microalgae samples.

**Figure 2:**
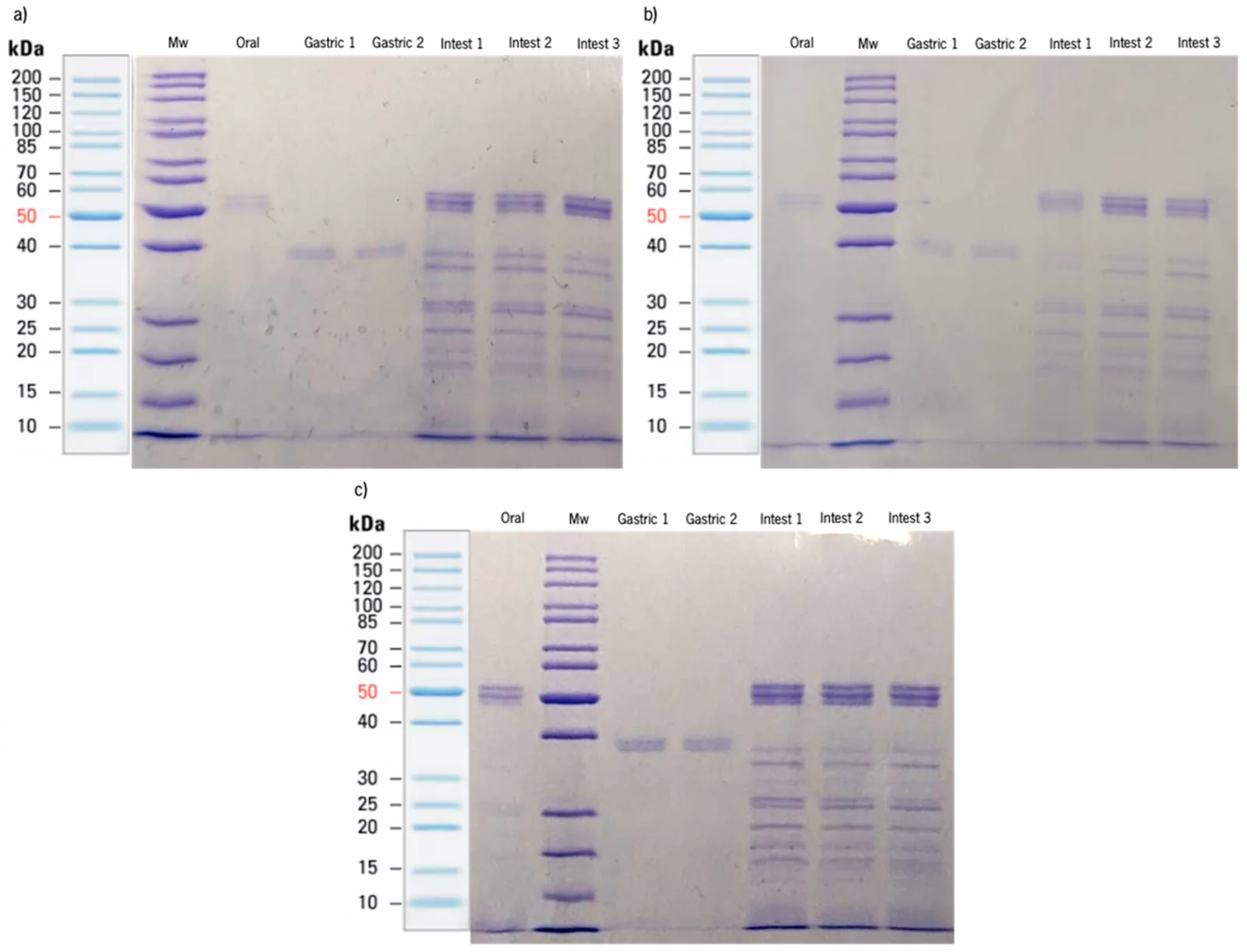
Electrophoresis gels from GI digestion samples: (**a**) untreated microalgae (Control); (**b**) microalgae with PEF pretreatment; and (**c**) samples from the GI enzymes as a blank control. Mw—molecular weight marker; oral—oral phase; Gastric 1—gastric phase after 60 min; Gastric 2—gastric phase after 120 min; Intest 1—intestinal phase after 20 min; Intest 2—intestinal phase after 120 min; Intest 3—intestinal phase after 150 min.

The increase in fluorescence intensity observed during GI digestion is likely attributable to the sequential addition of digestive enzymes, which contribute additional tryptophan-containing proteins to the system. This interpretation is supported by the similar fluorescence profiles obtained for enzyme controls. Furthermore, protein hydrolysis and conformational rearrangements occurring during digestion may alter the local environment of tryptophan residues, potentially reduce quenching effects or increase their exposure to more hydrophobic microenvironments, thereby contributing to the enhanced fluorescence signal. To further evaluate the changes, the fluorescence spectra were integrated to determine the total area under each peak (see Figure S1 in Supplementary Information). Figure S1 confirms a progressive increase in the integrated area observed throughout digestion, consistent with the sequential addition of GI enzymes. Interestingly, except for the oral phase and the intestinal stage at 20 min, PEF-treated samples showed consistently higher average fluorescence values compared to the control, although these differences were not statistically significant (*p* > 0.05). Overall, intrinsic fluorescence analysis alone was not suitable for detecting differences in protein content or digestibility between treatments, as the signal was largely dominated by GI enzymes, masking the possible contribution of microalgal proteins. The electrophoresis by SDS-PAGE was also performed to further evaluate protein profiles and assess potential differences in molecular weight distribution during digestion. SDS-PAGE was performed for all the microalgae samples collected during the GI digestion (Figure 2).

The SDS–PAGE gels obtained for the different samples exhibited highly similar protein profiles (Figure 2a–c). Most of the bands observed in Figure 2c were mainly attributed to the GI enzymes used during digestion. Likewise, the electrophoretic profiles of samples containing microalgae proteins, with or without PEF pretreatment, closely resembled both each other and the GI enzymes control. For example, α-amylase, used during the oral phase, has a molecular weight of approximately 50 kDa, whereas pepsin, employed during the gastric phase, has a molecular weight of around 34 kDa [27]. In addition, pancreatin used in the intestinal phase is a complex enzymatic mixture containing several digestive enzymes. Consequently, the electrophoretic profiles were largely dominated by proteins originating from the digestion fluids. Furthermore, enzymatic hydrolysis during digestion may have generated low-molecular-weight peptides that were not efficiently resolved or stained by SDS–PAGE. Given the limited ability of both SDS–PAGE and intrinsic fluorescence to discriminate microalgal proteins from digestive enzymes, the Lowry assay was subsequently employed to quantify total soluble protein.

**Figure 3:**
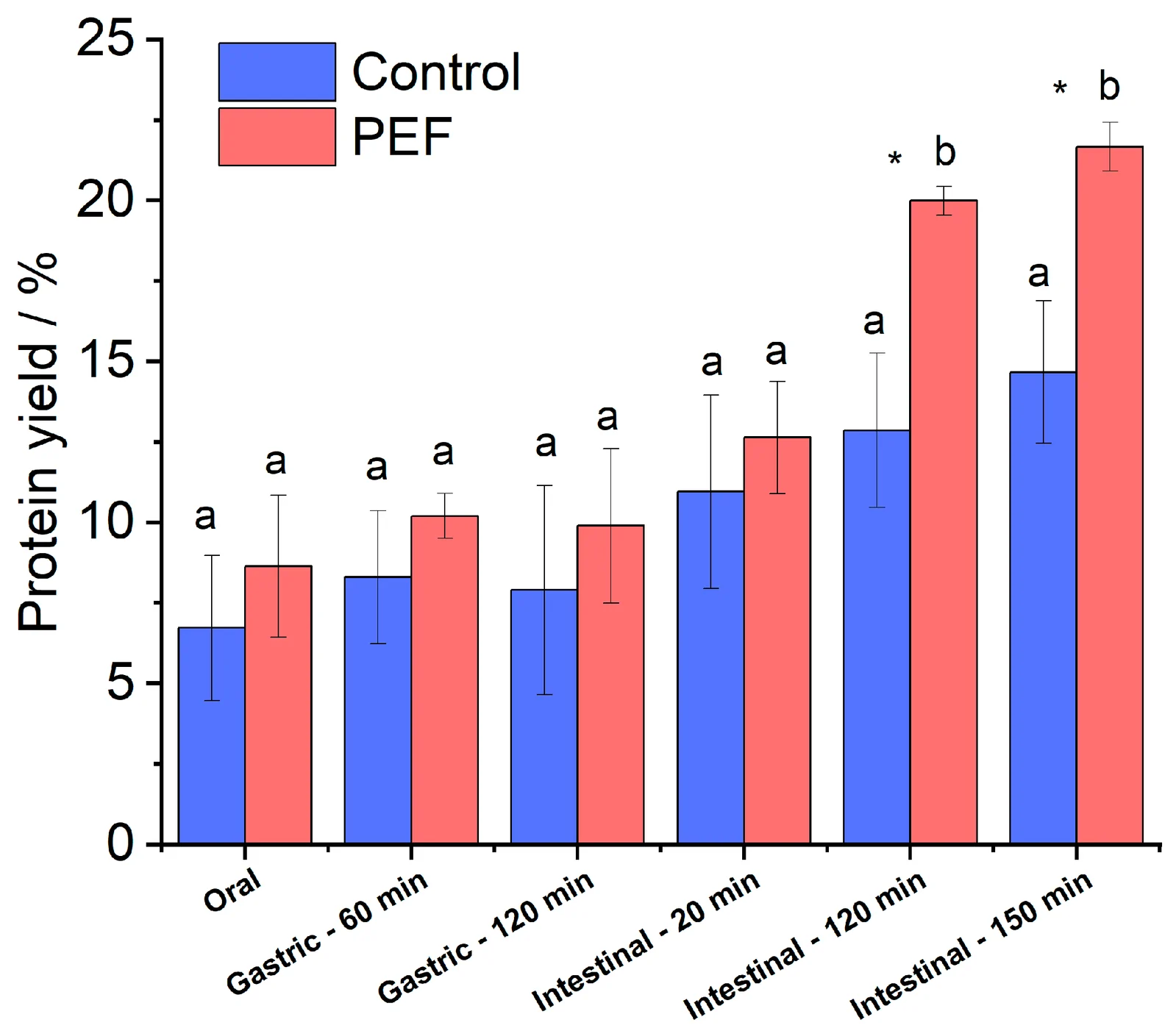
Total protein yield in untreated (control) and PEF-treated samples at each stage of the in vitro GI digestion: oral phase; gastric phase at 60 and 120 min; and intestinal phase at 20, 120, and 150 min. Different letters above the bars indicate statistically significant differences (*p* < 0.05) between digestion phases within the same treatment (Tukey post hoc test). The asterisks (*) indicate statistically significant differences between control and PEF at the same digestion phase. Results are expressed as mean ± SD (*n* = 3).

The Lowry method enabled the detection of differences in protein availability, as it quantifies total soluble protein regardless of partial degradation or the presence of exogenous digestive enzymes [28]. This is consistent with previous studies reporting the suitability of the Lowry assay for measuring both intact proteins and smaller peptides, as it is less dependent on molecular size and relatively robust in the presence of interfering substances or processing-related effects, such as heating or shear stress [29,30]. Therefore, the results presented in Figure 3 allow a direct comparison between digested samples, with PEF pretreatment representing the sole experimental variable.

Throughout most of the digestion process, no significant differences in soluble protein concentration were observed between the control and PEF-treated samples during the oral, gastric, and intestinal (<120 min) phases (*p* > 0.05). Nevertheless, a consistent trend toward higher protein release was evident in the PEF-treated samples from the onset of digestion, which likely contributed to the cumulative difference observed at later stages. These differences became statistically significant (*p* < 0.05) after 120 and 150 min of intestinal digestion. The latter sampling point corresponded to the completion of the digestion process (approximately 4 h), followed by an additional 30 min before final collection for analysis. This progressive increase in protein concentration may be correlated with the gradual increase in intrinsic fluorescence intensity, potentially due to a reduction in quenching effects promoted by protein digestion, as discussed previously.

The freeze-dried *C. vulgaris* used in this study contained a total protein content of 489 mg per g of dry cells, corresponding to approximately 50% protein per dry.

During the oral phase, 6.72 ± 2.25% of total proteins were released from untreated cells, whereas PEF-pretreated cells exhibited a higher release (8.63 ± 2.20%), indicating enhanced protein accessibility at this early stage as a result of electroporation. During the first 60 min of the gastric phase, protein release further increased to 8.30 ± 2.06% in the control samples and to 10.20 ± 0.70% in the PEF-treated samples, reinforcing the positive effect of PEF pretreatment on protein bioaccessibility. After 120 min of gastric digestion, no further increase was observed; protein availability was 7.90 ± 3.24% for the control and 9.90 ± 2.40% for the PEF-treated cells. At this stage, proteins are exposed to harsh acidic conditions (pH ≈ 3) and pepsin activity present in the gastric fluid, leading to proteolysis and peptide formation [31,32]. These processes may explain the slight decrease in detectable protein content, as hydrolyzed peptides can be underestimated by the Lowry method [33,34]. Nevertheless, across the entire gastric phase, PEF-treated samples showed a tendency toward higher protein release, although the differences against untreated samples were not statistically significant (*p* > 0.05). This trend may indicate improved enzyme accessibility following PEF-induced cell permeabilization, but further evidence would be required to confirm this effect. After 20 min of the intestinal phase, a slight increase in total protein was observed in both treated and untreated samples, although differences were not yet statistically significant. However, by the end of the intestinal phase, clear and significant differences emerged between treatments.

After 120 min of intestinal digestion, the protein bioaccessibility reached 12.85 ± 2.39% in the control and 19.98 ± 0.45% in PEF-treated samples. At the final sampling point (150 min of intestinal digestion), protein bioaccessibility was significantly higher (*p* < 0.05) in PEF-pretreated biomass (21.66 ± 0.76%) compared to the untreated control (14.66 ± 2.21%), representing an increase of approximately 47%. The fact that the effect of PEF became more pronounced during the intestinal phase suggests that the treatment did not necessarily cause extensive immediate protein release but rather increased the accessibility of intracellular proteins to digestive enzymes. This interpretation is consistent with the well-established observation that physical and chemical disruption of microalgal cell walls can improve digestibility when whole cells are consumed [35]. In *C. vulgaris*, this effect may be particularly relevant because its rigid cell wall can restrict the diffusion of digestive enzymes and limit the release of intracellular proteins. Species such as *C. vulgaris*, which possess a complex and rigid cell wall structure, exhibit lower digestibility compared to species with less complex walls, such as *Arthrospira platensis* [36]. Specifically, the presence of cellulose, hemicellulose, and glycoproteins could be the reason for the cell wall resistance to digestion; the absence of these structures could increase the digestibility by 15 times [37,38,39].

Accordingly, the limited differences observed during the earlier digestion stages may reflect insufficient enzymatic accessibility to generate a measurable treatment effect, whereas prolonged exposure to intestinal enzymes may have amplified the advantage provided by PEF-induced cell permeabilization. Previous studies have also demonstrated the importance of pretreatment strategies before digestion. For example, Kose et al. showed that crude *C. vulgaris* biomass exhibited approximately 35% digestibility, whereas enzymatic pretreatment with pancreatin doubled this value. Although protein release during simulated GI digestion was not reported, this study supports the relevance of pretreatment approaches to improve nutrient accessibility [10].

In the present work, PEF likely induced structural alterations in the cell wall and membranes of *C. vulgaris*, increasing cellular permeability and facilitating the penetration of digestive enzymes during digestion [6]. Given the intrinsic resistance of *C. vulgaris* cells to GI enzymatic degradation, largely due to their cellulose- and hemicellulose-rich cell wall structure [36], a pretreatment step may therefore be beneficial to improve nutrient accessibility prior to consumption. The findings provide evidence that PEF can be an effective approach for enhancing protein bioaccessibility by promoting cell permeabilization and improving the efficiency of GI enzymatic hydrolysis. Nevertheless, as changes in cell permeability and cell-wall structure were not directly monitored during digestion in the present study, the proposed mechanism should be regarded as a plausible interpretation rather than a demonstrated causal relationship.

### 3.2. Microalgae Amino Acids Profile

**Figure 4:**
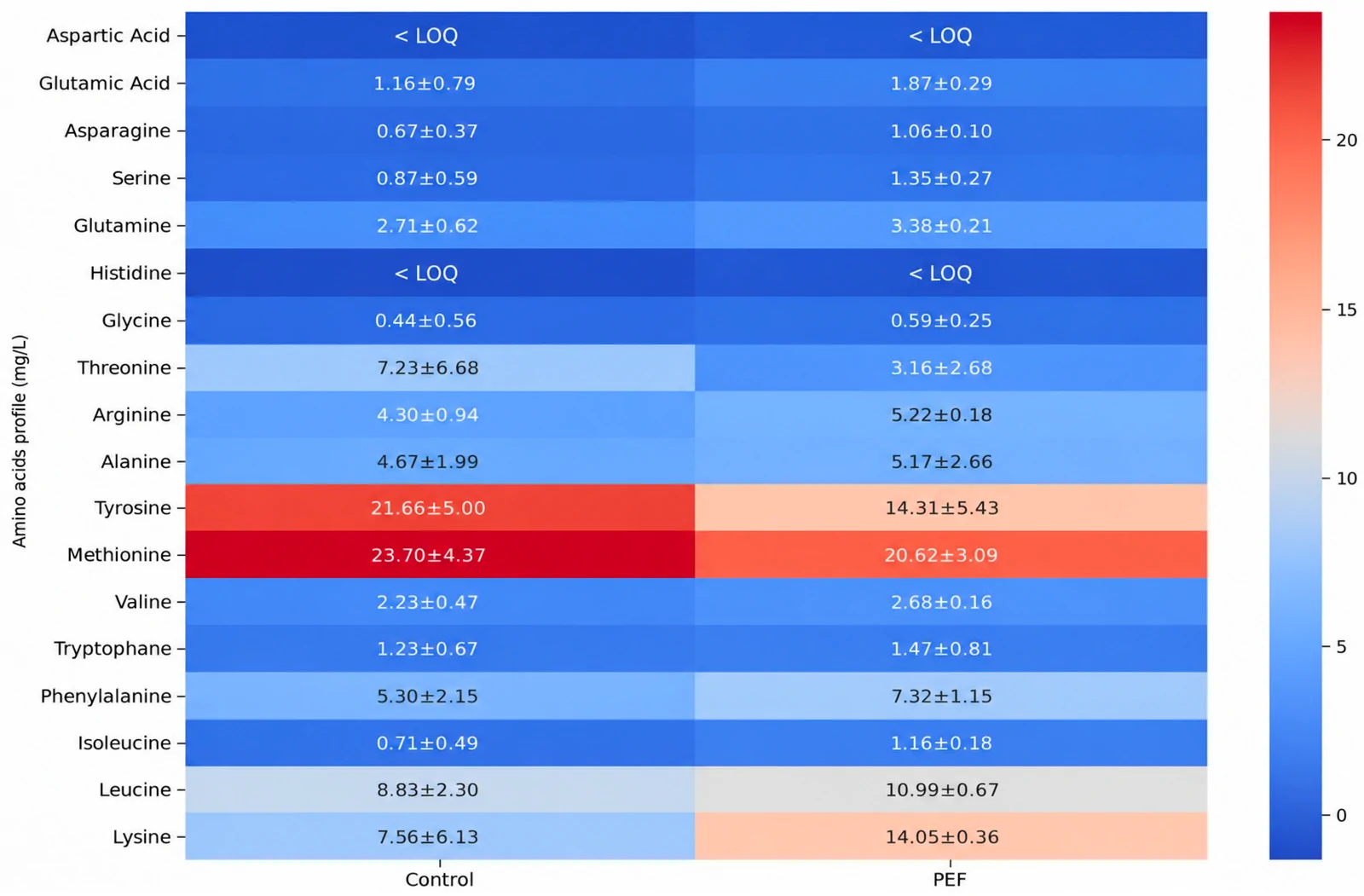
Amino acid profile of *Chlorella vulgaris* after in vitro GI digestion. Results are expressed as mean ± SD (*n* = 3). No statistically significant differences (*p* > 0.05) were found between Control and PEF-treated samples. LOQ, limit of quantification.

The final digested products from the in vitro GI digestion were analyzed to characterize the amino acid profile of the microalga. This analysis aimed to identify the amino acids potentially available for absorption following digestion. Figure 4 presents the amino acid profiles of *C. vulgaris* after digestion, comparing untreated and PEF-treated cells. A control sample containing only GI enzymes was also analyzed, and its amino acid values were used to correct for enzymatic interference in the samples containing the microalgal cells.

The amino acid profile was similar between both treated and untreated cells, indicating that PEF processing of *C. vulgaris* does not promote the selective release or degradation of specific amino acids. No statistically significant differences were observed between treated and untreated microalgae (*p* > 0.05). Among the detected amino acids, tyrosine and methionine were present at relatively higher concentrations under both conditions. In untreated samples, tyrosine and methionine concentrations were 21.7 ± 5 mg·L^−1^ and 23.7 ± 4.37 mg·L^−1^, respectively, whereas in PEF-treated samples their concentrations were 14.3 ± 5.43 mg·L^−1^ and 20.6 ± 3.1 mg·L^−1^, respectively.

A closer examination analysis of the heat map suggests a slightly higher relative abundance of certain amino acids in PEF-treated cells. For instance, Lysine exhibited higher levels following treatment. Although these differences were not statistically significant (*p* > 0.05), several amino acids appeared in moderately higher amounts in PEF-treated samples, suggesting a potential enhancement in their release from the cellular matrix, corroborating previous findings.

Tibbets et al. quantified specific amino acids in another strain of *C. vulgaris*, reporting tyrosine and lysine contents of 2.41 ± 0.0% (combined with phenylalanine) and 1.42 ± 0.02% of dry weight, respectively [40]. These values differ from those obtained for *C. vulgaris* CCAP 211/11B used in the present study.

The amino acid compositions of *Chlorella vulgaris* vary considerably across the literature. In one study, leucine was reported as one of the most abundant essential amino acids (2.05 ± 0.03% of dry weight), whereas in the present work its concentration was below 10 mg·L^−1^ in untreated samples. Safi et al. identified aspartic acid and alanine as the predominant amino acids (10.09 ± 0.02 and 10.05 ± 0.03 g per 100 g biomass, respectively), while these were not detected in relevant amounts in our samples [41].

Similarly, Pinto et al. reported glutamic acid, histidine, and arginine as the most abundant amino acids in *C. vulgaris* grown in saline medium [42]; however, these amino acids were not predominant in our samples, which were grown under non-saline conditions [42]. Ursu et al. and Spínola et al. also described glutamic acid as one of the major amino acids, with lysine and leucine representing approximately 9.46% and 9.87%, respectively [43,44]. In contrast, glutamic acid showed one of the lowest concentrations among the quantified amino acids in the present study, and tyrosine and methionine were not detected at low levels, contrary to some literature reports. Despite differences in absolute concentration, the overall qualitative amino acids identified in this study align with previous reports. The observed variability appears to be primarily quantitative rather than qualitative and is largely influenced by cultivation conditions (i.e., growth phase and medium composition) as well as the analytical methodologies employed. Furthermore, the results indicate that the PEF conditions applied in this study did not induce amino acid degradation, suggesting that electrochemical reactions, excessive Joule heating, or radical formation were effectively minimized.

### 3.3. Viability Assays of the Digested Microalgae

At the end of the in vitro GI digestion, cell viability assays were performed using the Caco-2 human intestinal cell line to evaluate potential beneficial or cytotoxic effects of the digested microalgae biomass.

One of the main concerns when working with electric field-based technologies in the food industry is the direct contact between the food matrix and the electrodes. This concern arises from electrochemical reactions that may occur at the electrode-product interface, particularly in liquid systems such as the microalgae suspensions treated with PEF. These reactions involve electron transfer between electrodes and surrounding medium and have been widely described in the literature. Electrode corrosion during treatment may lead to the release of metal ions into the processed sample, potentially increasing the toxicity of the final product [45,46,47]. Previous studies have shown that direct contact between stainless steel electrodes and food matrices during electric field processing can result in metal migration into the medium [24,46]. The extent of this migration depends on factors such as electrode composition and processing parameters, including field strength, frequency, and treatment time. Therefore, evaluating the potential toxicological impact associated with metal release is essential when applying electro-technologies to food systems.

**Figure 5:**
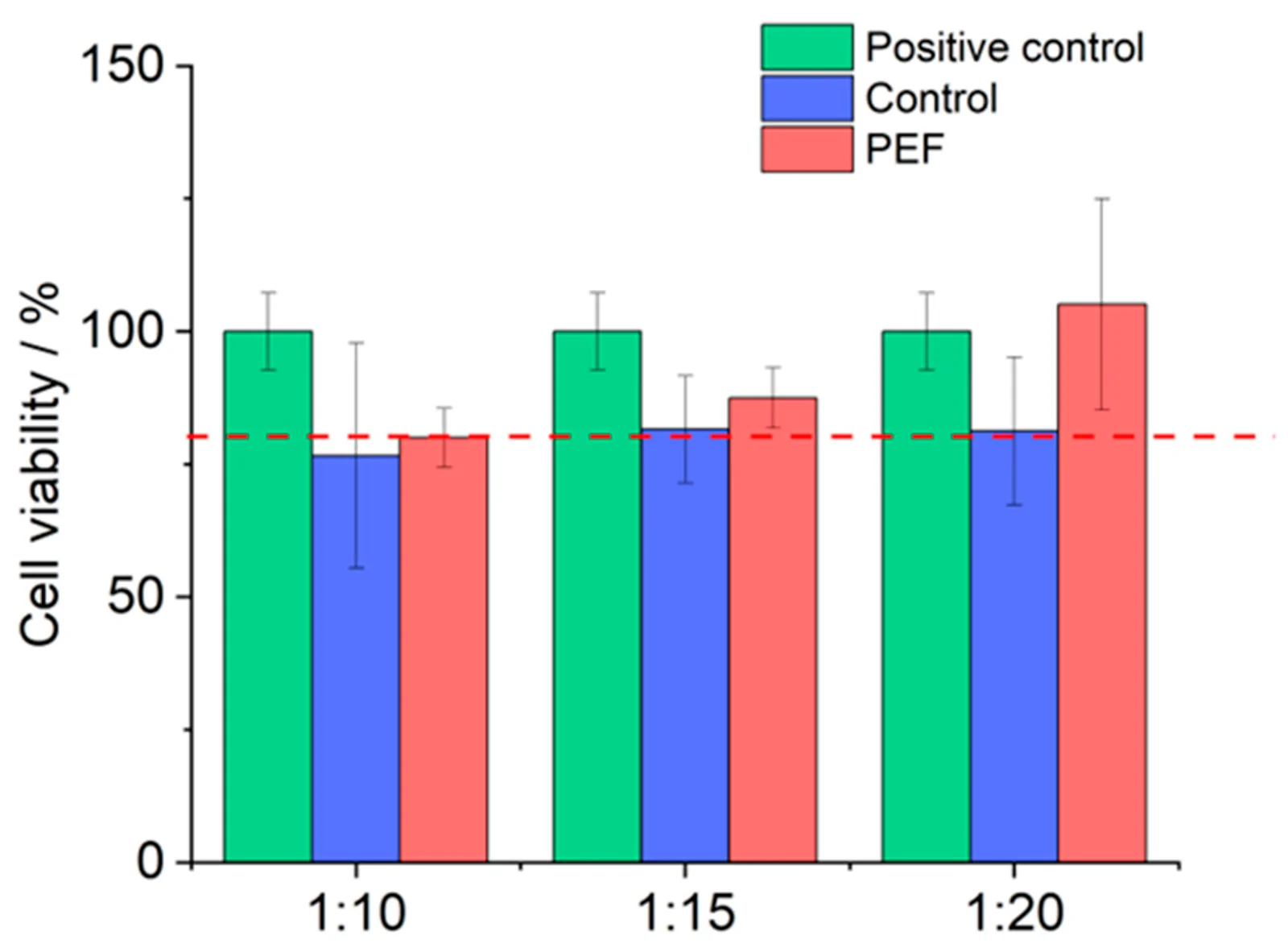
Caco-2 cell viability after exposure to different concentrations (1:10, 1:15, 1:20 v·v^−1^) of untreated or PEF-treated microalgae extracts following GI digestion. The results are presented as mean ± SD (*n* = 4). The red dotted line corresponds to 80% of cell viability. No statistically significant differences (*p* > 0.05) were found between the control, PEF-treated samples, and positive control. The vertical error bars represent the standard deviation (SD).

Figure 5 presents the viability of Caco-2 cells after exposure to the digested microalgal biomass samples. Cell viability values below 80% are generally considered indicative of cytotoxicity [48]. In the present study, relatively high variability was observed among biological replicates, as reflected by the standard deviations, and the results should therefore be interpreted with caution. Although most mean cell viability values were close to or above 80%, some conditions showed mean values below this threshold. Overall, however, PEF-pretreated samples did not show a consistent decrease in cell viability compared with the corresponding controls. These results suggest that, under the selected processing conditions, PEF pretreatment did not produce a clear increase in the cytotoxic effects of digested *C. vulgaris* biomass.

The MTT assay showed viability values of 76.63 ± 21.13%, 81.56 ± 10.10%, and 81.24 ± 13.92% for untreated microalgae at dilutions of 1:10, 1:15, and 1:20 respectively. Similarly, PEF-pretreated samples showed viability values of 79.96 ± 5.51%, 87.47 ± 5.75%, and 105.08 ± 19.83% at the same dilutions. Overall, no relevant toxic effects were observed in the Caco-2 cell line.

Microalgal proteins are widely recognized for their potential beneficial effects on human and animal health. Guo et al. also reported the absence of cytotoxic effects on Caco-2 cells exposed to microalgal extracts, although their study evaluated extracts without simulating the complete digestion process [49]. They concluded that microalgae extracts may improve gut health and protect the intestinal barrier against inflammatory reactions [49]. Similarly, Magpusao et al. investigated *Arthrospira* and *Nannochloropsis* pretreated with high-pressure homogenization (HPH) and observed protective effects against oxidative stress in Caco-2 cells, highlighting the bioprotective potential of microalgal proteins against reactive oxygen species [50].

## 4. Conclusions and Future Perspectives

PEF pretreatment enhanced protein bioaccessibility after in vitro GI digestion compared to untreated *C. vulgaris*. Based on the known mechanisms of PEF, the treatment likely induced partial cell disruption and/or electroporation, increasing membrane permeability and thereby improving the accessibility of digestive enzymes to intracellular proteins. These results emphasize the strategy of applying mild physical pretreatments when microalgal biomass is used in its whole form to improve bioaccessibility of its nutrients. PEF-pretreated *C. vulgaris* exhibited protein bioaccessibility values above 21%, highlighting PEF as a promising strategy to improve microalgal digestibility and protein availability. However, intrinsic fluorescence spectroscopy and SDS-PAGE did not confirm the differences observed with the Lowry assay. This discrepancy is likely related to the complexity of the digested matrix, where microalgal proteins, hydrolysis products, digestive enzymes, and other digestion components coexist and may interfere with protein quantification and structural analysis. In particular, extensive proteolysis during digestion reduces the presence of intact protein bands detectable by SDS-PAGE, while fluorescence signals may be affected by overlapping contributions from peptides, aromatic amino acids, and digestive enzymes.

The amino acid profiles obtained after GI digestion were comparable between untreated and PEF-treated cells, suggesting that PEF treatment—and any potential electrochemical effects—did not alter the qualitative amino acid composition, but rather influenced their overall release and bioaccessibility. Furthermore, cytotoxicity assays performed on Caco-2 human intestinal cells showed no adverse effects associated with PEF-pretreated microalgae, supporting the safe application of this technology for microalgal processing.

A limitation of the present study is that only one PEF treatment condition (20 kV·cm^−1^, 100 kJ·kg^−1^) was evaluated. Although this condition was selected based on previous work and was shown to enhance protein bioaccessibility without adverse effects in Caco-2 cells, the present experimental design does not allow for a systematic assessment of the effects of PEF intensity or specific energy input on protein release, bioaccessibility, or cytotoxicity. Future studies should therefore investigate a broader range of PEF treatment conditions to determine potential dose-dependent effects and to identify treatment windows that maximize protein bioaccessibility while maintaining cellular safety, minimizing unnecessary energy input, and preserving other valuable microalgal components.

Overall, these findings highlight both the analytical challenges associated with protein assessment after in vitro digestion and the relevance of appropriate pretreatment strategies to enhance protein bioaccessibility. This is particularly important for microalgae with rigid and structurally complex cell walls, such as *C. vulgaris*, where limited digestibility can restrict the release and availability of intracellular proteins.

## Data Availability

The original contributions presented in this study are included in the article/Supplementary Materials. Further inquiries can be directed to the corresponding authors.

## Supplementary Materials

The following supporting information can be downloaded at https://www.mdpi.com/article/10.3390/foods15213882/s1, Figure S1: Peak integration area from the intrinsic fluorescence spectra of untreated (Control) and PEF-pretreated samples at every stage of GI digestion. The results are expressed as a mean ± SD (*n* = 3), and Origin Pro 2018 software was used for the integration of each peak. The results obtained from the integration were exported to GraphPad Prism for the statistical analysis with a Two-Way ANOVA with a confidence level of 95%. ns: non-significant; Table S1: Preparation of derivatization reagents and mobile phases.
