# Supplementary material for: Application of Pulsed Electric Fields to Enhance the Digestive Bioaccessibility of Chlorella vulgaris Protein Fractions
Source: Foods. 2026 Oct 29;15(21):3882. doi: 10.3390/foods15213882 (PMC13763361; doi:10.3390/foods15213882)
Supplement: Supplementary file 1 [file foods-15-03882-s001.zip › foods-4578100-supplementary.pdf]

Application of Pulsed Electric Fields to Enhance the Digestive Bioaccessibility of Chlorella vulgaris Protein Fractions

Luís Machado[1], Serena Carpentieri[2], Raquel F.S. Gonçalves[1,3], Joana T. Martins [1,3], Maria Silva[1], José A. Teixeira[1,3], Giovanna Ferrari[2,4], Ricardo N. Pereira[1,3+] and Gianpiero Pataro[2]

[1]CEB—Centre of Biological Engineering, University of Minho, 4710-057 Braga, Portugal;

[2] Department of Industrial Engineering, University of Salerno, Via Giovanni Paolo II, 132 – 84084 Fisciano (SA), Italy

[3]LABBELS—Associate Laboratory, Braga, Guimarães, Portugal

[4]ProdAl Scarl – University of Salerno, Via Giovanni Paolo II, 132 – 84084 Fisciano (SA), Italy

Supplementary information

Materials and methods

Supplementary Table S1. Preparation of derivatization reagents and mobile phases

Reagents and Solutions	Preparation
Mercaptopropionic acid (MPA) Solution	Dissolve 10 µL of 3-mercaptopropionic acid in 10 mL of 0.1N borate buffer
o-Phthalaldehyde (OPA) Solution	Add 0.3 mL of ethanol in 10 mg of o-Phthalaldehyde until dissolves completely. Then, add 0.7 mL of 0.1N borate buffer and 4 mL of ultrapure water.
MPA/OPA solution	Mix 600 µL of MPA Solution and 300 µL of OPA Solution
Fluorenylmethyl chloroformate (FMOC) solution	Dissolve 10 mg of 9-fluorenylmethyl chloroformate in 100 mL of acetonitrile
Phosphoric acid (PA) Solution	Add 0.5 mL of phosphoric acid (85 %) in 100 mL of ultrapure water
Borate buffer 0.1M	Dissolve 0.62 g of boric acid and 0.2 g sodium hydroxide in 100 ml of ultrapure water
Eluent A	Dissolve 2.67 g of sodium acetate tri-hydrate and 41 µL of acetic acid in 1000 µL of ultrapure water

Eluent B	Mix 900 mL of acetonitrile and 100 mL of ultrapure water
Eluent C	Dissolve 0.19 g of EDTA-2NA, 2.03 g of sodium acetate trihydrate, and 308 μL of acetic acid in 1000 mL of ultrapure water
Eluent D	1000 mL of ultrapure water

Results and discussion

To further support and confirm the protein quantification results obtained by the Lowry method, fluorescence spectroscopy analyses and SDS-Page were performed on the digested microalgae. Due to the high variability of the obtained results and strong interference of the gastrointestinal enzymes, those results will be mentioned in this Supplementary Material.

Fluorescence spectroscopy of the digested microalgae

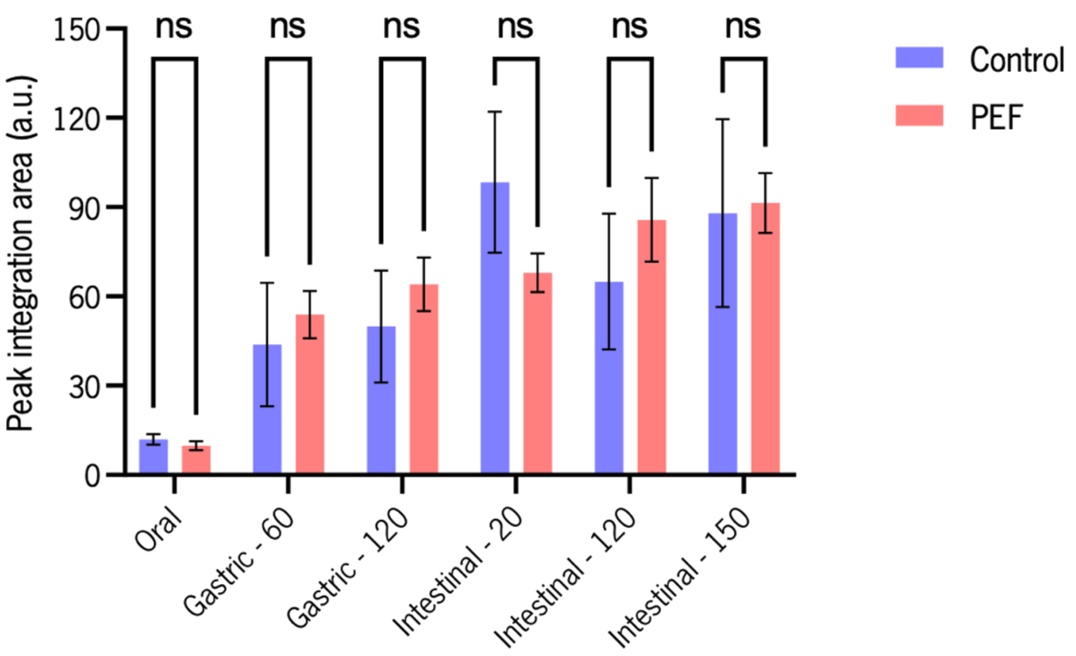

Figure S1. Peak integration area from the intrinsic fluorescence spectra of untreated (Control) and PEF-pretreated samples at every stage of GI digestion. The results are expressed as a mean ± SD (n=3), and Origin Pro 2018 software was used for the integration of each peak. The results obtained from the integration were exported to GraphPad Prism for the statistical analysis with a Two-Way ANOVA with a confidence level of 95%. ns: non-significant.

To better understand the obtained results from the fluorescence spectra, all the spectra were integrated to determine the total area of each peak. Figure S1 shows the total area of each spectrum at each moment of the digestion. It is possible to observe a constant increase in the area, which is explained by the addition of more GI enzymes during the digestion process. Interestingly, except for oral and the intestinal stage (20 min), the PEF-treated samples showed average fluorescence values consistently higher than the control, although no statistically significant differences were observed. This trend agrees with the
